# Biologic Underutilization and Healthcare Burden in US Patients with Type 2 Severe Asthma

**DOI:** 10.2147/JAA.S580937

**Published:** 2026-04-22

**Authors:** Jihye Park, Steven Gelwicks, Loretta A Jacques, David Muccino, Justin Kwiatek, Rafael Alfonso-Cristancho, Victoria S Benson

**Affiliations:** 1 Epidemiology, Global Regulatory, Safety & Quality, GSK, Collegeville, PA, USA; 2 Real World Analytics, GSK, Collegeville, PA, USA; 3 Clinical Science Respiratory, GSK, London, UK; 4 Clinical Science Respiratory, GSK, Collegeville, PA, USA; 5 US Medical Affairs, GSK, Collegeville, PA, USA; 6 Global Real-World Evidence and Health Outcomes Research, GSK, Collegeville, PA, USA; 7 Global Epidemiology, Organization of the Chief Medical Officer, GSK, London, UK

**Keywords:** biologics, population analysis, respiratory, therapeutics

## Abstract

**Background:**

Detailed real-world evidence on the unmet needs and economic burden of patients with type 2 severe asthma, particularly in the biologic therapy era, remains limited.

**Purpose:**

To characterize unmet treatment needs and economic burden in patients with type 2 severe asthma and a history of frequent exacerbations who could benefit from biologic therapy.

**Patients and Methods:**

This retrospective cohort study used healthcare claims data from the US Optum’s de-identified Clinformatics^®^ Data Mart database (January 1, 2018–December 31, 2021). Patients with type 2 severe asthma were included if they received Global Initiative for Asthma (GINA) step 4 (GINA 4)/5 treatment, experienced ≥2 exacerbations, and had a blood eosinophil count ≥150 cells/μL in the baseline period (ie. 12 months prior to the index date [study entry]). Analyses were descriptive.

**Results:**

Overall, 2827 patients met the study definition for type 2 severe asthma (GINA 4: n=1513; GINA 5: n=1314), with 11.9% of patients (GINA 4: 5.2%; GINA 5: 19.6%) receiving biologics at baseline. Systemic/oral steroid-defined exacerbations were most common at baseline and follow-up across GINA steps. Overall, similar proportions of patients at GINA 4 and 5 (71.6% and 70.5%, respectively) experienced ≥1 exacerbation during the follow-up period. The mean (SD) total number of exacerbations was 1.6 (1.8) at GINA 4 versus 1.7 (1.9) at GINA 5. Mean (SD) steroid-defined exacerbations were 1.3 (1.5) versus 1.4 (1.6), emergency-department (ED)-defined exacerbations were 0.2 (0.9) versus 0.2 (0.8), and hospitalization-defined exacerbations were 0.04 (0.3) and 0.06 (0.3) in the respective treatment groups. Patients at GINA step 5 receiving biologic therapies at baseline had lower asthma-related inpatient admissions costs versus those not receiving biologic therapies.

**Conclusion:**

Patients with type 2 severe asthma at GINA steps 4/5 experience similar disease burden. These findings highlight a potential unmet need for earlier phenotypic assessment in this population.

## Introduction

Patients with severe asthma may be categorized as being at Global Initiative for Asthma (GINA) treatment step 4 or 5 of asthma management, based on the medications they are currently receiving, as a proxy for the level of severity. Patients receiving medium-/high-dose maintenance combination inhaled corticosteroids (ICS) and long-acting β_2_-agonists (LABA) are considered to be at GINA treatment step 4,^1^ while patients receiving this treatment plus add-on long-acting muscarinic antagonist (LAMA) therapy are considered to be at GINA treatment step 5.^1^ If asthma remains uncontrolled (ie, with patients experiencing either poor symptom control or frequent/serious exacerbations unresponsive to standard of care), use of low-dose oral corticosteroids (OCS) may also be considered.^1^,^2^ Physicians may alternatively consider whether the patient might benefit from biologics; at treatment step 5, GINA recommends phenotypic characterization of patients with severe uncontrolled asthma for potential biologic therapy (including anti-interleukin-4 receptor α [IL-4Rα], anti-IL-5, and anti-IL-5R therapies).^1^

Type 2 inflammation is driven by core cytokines such as IL-4, IL-5, and IL-13, and is the underlying pathology for more than 80% of people with severe asthma (ie, type 2 severe asthma), leading to recurring symptoms and unpredictable exacerbations.^1^,^3–6^ Targeting the core cytokines IL-4, IL-5, and IL-13 (or their receptors) via biologics can mitigate the effects of chronic type 2 inflammation, ultimately supporting patients in preventing exacerbations and achieving clinical remission.^7–13^

Despite the GINA recommendation regarding treatment options for severe asthma, underutilization of asthma biologics and delays in their initiation remain common in real-world clinical settings.^14^ Biologic initiation and adherence are influenced by factors such as insurance type, prior authorization and availability of phenotyping.^15–17^ Delayed biologic initiation or underutilization can lead to inconsistent control of inflammation and increase the risk of exacerbations and disease progression.^18^ There is limited evidence on the unmet needs and economic burden of patients with type 2 severe asthma who might be eligible for biologic therapy, as well as on the disease burden among patients at GINA treatment steps 4 and 5. Therefore, the aim of this analysis was to characterize unmet treatment needs and the economic burden in patients with type 2 severe asthma and a history of frequent exacerbations in real-world settings who could benefit from biologic therapy.

## Materials and Methods

### Study Objectives

The objective of this study was to describe demographic, clinical, treatment, healthcare resource utilization (HCRU), and cost characteristics of adult patients with type 2 severe asthma in real-world clinic settings in the USA. Topline results from this population have previously been published.^19^,^20^

### Study Design

**Figure 1:**
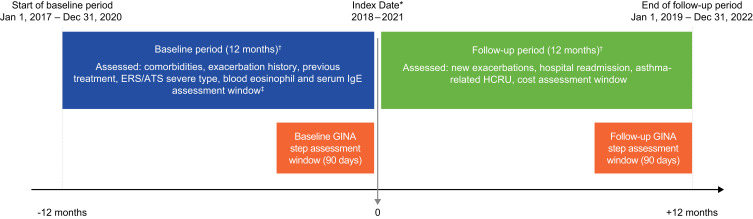
Study design. *≥1 medical claim with an asthma medical code in any position during the cohort definition period. If there were multiple asthma claims during the cohort definition period, the earliest service date in which all other criteria were met, served as the index date; ^†^Up to 30-day gaps in medical or pharmacy enrollment were allowed; ^‡^If a patient had >1 value during the study period, the maximum value was used.

**Abbreviations**: ATS, American Thoracic Society; ERS, European Respiratory Society; GINA, Global Initiative for Asthma; HCRU, healthcare resource utilization; IgE, immunoglobulin E.

This retrospective cohort study (GSK Study ID: 220237) utilized healthcare claims data from the US Optum’s de-identified Clinformatics^®^ Data Mart Database (Optum^®^ CDM), which is derived from a database of administrative health claims for members of large commercial and Medicare Advantage health plans.^21^ The analysis included individuals with a diagnosis of asthma (International Classification of Diseases, Tenth Revision, Clinical Modification [ICD-10-CM]: J45xx) between 2018 and 2021. Individuals were indexed on their first diagnosis and required to have a record of asthma medication dispensed within the 90 days prior to the index diagnosis date. The individuals were also required to have at least 12 months of continuous enrollment prior to the earliest study entry date for assessment of baseline characteristics, and at least 12 months of continuous enrollment after the latest study entry date for follow-up (Figure 1).

### Study Population

Eligible patients were adults (≥18 years) with an asthma diagnosis who were receiving asthma treatment (as described above). Patients had continuous medical and pharmacy insurance coverage for ≥12 months prior to the index date. Patients included in the analytic cohort of type 2 severe asthma were in receipt of GINA step 4/5 in the 90 days prior to the index diagnosis, had experienced ≥2 exacerbations during the 12 months pre-index, and had a blood eosinophil count of ≥150 cells/μL during the same period. It should be noted that patients were categorized into GINA treatment step 4/5 based on their asthma treatment in the 90 days prior to the index date. Patients were excluded from this study if they had a diagnosis of active respiratory tuberculosis, lung cancer, or cystic fibrosis (defined as ≥1 medical claim with a relevant code in any position during the study period).

### Data Source, Variables, and Analysis

The Optum^®^ CDM database served as the source for claims data included in this analysis. Variables of interest included demographics and clinical characteristics at baseline, and clinical characteristics, asthma-related HCRU and expenditure during follow-up. All demographic, clinical, treatment, HCRU and cost characteristics data were analyzed descriptively. All between-group comparisons are purely descriptive. Further details are provided in the Supplementary Methods.

### Ethics

The Optum^®^ CDM is statistically de-identified under the Expert Determination method consistent with the Health Insurance Portability and Accountability Act (HIPAA) and managed according to Optum customer data use agreements. Optum^®^ CDM administrative claims submitted for payment by providers and pharmacies were verified, adjudicated and de-identified prior to inclusion. This study complied with all applicable US laws (ie, HIPAA) regarding subject privacy, and no direct patient contact or primary collection of individual patient data occurred. Since this was an observational study using de-identified patient-level data from healthcare databases, reported only in aggregate, it did not require ethics committee approval or informed consent from patients.

## Results

### Study Attrition

Of the 2,378,445 patients with ≥1 asthma diagnosis medical claim between January 1, 2018, and December 31, 2021, a total of 2827 adult patients (≥18 years) met all the study criteria and were identified as having type 2 severe asthma. Overall, 1513 patients were classified as being at GINA treatment step 4 and 1314 at GINA treatment step 5 (eFigure 1). It should be noted that patients who were prescribed a biologic during the 1-year baseline period and then discontinued the biologic within the 90-day period (n=75) were classified as GINA treatment step 4.

### Baseline Patient Characteristics

Baseline patient characteristics for the overall population as well as by GINA steps 4 and 5 subgroups are shown in Table 1. The majority of patients were female (74.0%), aged over 60 years (median [Q1, Q3] 65 [54, 72] years) and had comorbid conditions, with 66.4% of all patients receiving Medicare. The most frequent select comorbidities were allergic rhinitis, atopy, general cerebrovascular disorders, and obesity; a lower proportion of patients at GINA treatment step 4 had allergic rhinitis and atopy compared with patients at GINA treatment step 5. Prevalence of these pre-existing comorbidities was lower in patients at GINA treatment step 4 versus 5. Exacerbation history was similar across GINA subgroups and predominantly driven by systemic/oral steroid-defined exacerbations for both. The majority of patients (90.7%) had previously received reliever medication (ie, short-acting β_2_-agonists [SABAs] or short-acting muscarinic antagonists [SAMAs]) or ICS/LABA combination therapy (88.0%). Overall, 96.1% of patients at GINA treatment step 4 received combination ICS/LABA therapy in the prior year, with patients also receiving triple therapy (25.6%), LAMA monotherapy (24.7%), leukotriene receptor antagonists (LTRAs) (59.2%) and maintenance OCS (37.3%) at varying rates. Comparatively fewer patients at GINA treatment step 5 had received combination ICS/LABA therapy in the same time period (78.6%), but a larger proportion had received triple therapy (32.7%), LAMA monotherapy (37.3%), LTRAs (68.4%) or maintenance OCS (50.8%; Table 1). It should be noted that patients in either GINA treatment step were potentially receiving other therapies concurrently. In the prior year, a lower proportion of patients at GINA treatment step 4, compared with step 5, had received biologic therapy (5.2% vs 19.6%). Baseline characteristics for patients at GINA treatment steps 4 and 5 stratified by receipt of biologic therapies are shown in eTable 1.

**Table 1:** Baseline Demographics and Clinical Characteristics in Patients with Type 2 Severe Asthma, Overall and by GINA Treatment Step, in the Prior 12 Months

	Patients with Type 2 Severe Asthma* at GINA Step 4 (n=1513)	Patients with Type 2 Severe Asthma* at GINA Step 5 (n=1314)	Overall Population of Patients with Type 2 Severe Asthma* (n=2827)
Female, n (%)	1141 (75.4)	952 (72.5)	2093 (74.0)
Age in years, mean (SD)	62.4 (14.4)	61.8 (14.4)	62.1 (14.4)
Race/ethnicity, n (%)			
White	874 (57.8)	793 (60.4)	1667 (59.0)
Black	229 (15.1)	205 (15.6)	434 (15.4)
Hispanic	272 (18.0)	214 (16.3)	486 (17.2)
Asian	56 (3.7)	27 (2.1)	83 (2.9)
Missing	82 (5.4)	75 (5.7)	157 (5.6)
Geographic region (USA), n (%)			
Northeast	164 (10.8)	143 (10.9)	307 (10.9)
North Central	132 (8.7)	115 (8.8)	247 (8.7)
South	822 (54.3)	707 (53.8)	1529 (54.1)
West	392 (25.9)	347 (26.4)	739 (26.1)
Missing	3 (0.2)	2 (0.2)	5 (0.2)
Education level, n (%)			
High school diploma	444 (29.3)	353 (26.9)	797 (28.2)
Less than Bachelor’s degree	767 (50.7)	703 (53.5)	1470 (52.0)
Bachelor’s degree or higher	225 (14.9)	190 (14.5)	415 (14.7)
Unknown/other	20 (1.3)	15 (1.1)	35 (1.2)
Index year, n (%)			
2018	977 (64.6)	827 (62.9)	1804 (63.8)
Post-2018	536 (35.4)	487 (37.1)	1023 (36.2)
Comorbidities (≥25% of population), n (%)			
Allergic rhinitis	780 (51.6)	768 (58.4)	1548 (54.8)
Anxiety	456 (30.1)	415 (31.6)	871 (30.8)
Atopy	806 (53.3)	792 (60.3)	1598 (56.5)
General cardiac disorders	672 (44.4)	602 (45.8)	1274 (45.1)
General cerebrovascular disorders	1071 (70.8)	956 (72.8)	2027 (71.7)
COPD^†^	675 (47.1)	608 (49.3)	1283 (48.1)
CRS with/without nasal polyps	437 (28.9)	437 (33.3)	874 (30.9)
CRS with nasal polyps	99 (6.5)	101 (7.7)	200 (7.1)
CRS without nasal polyps	338 (22.3)	336 (25.6)	674 (23.8)
Depression	426 (28.2)	347 (26.4)	773 (27.3)
GERD	710 (46.9)	682 (51.9)	1392 (49.2)
Obesity	922 (60.9)	793 (60.4)	1715 (60.7)
Quan-CCI, mean (SD)	3.0 (2.5)	3.1 (2.5)	3.0 (2.5)
CCI components (≥15% of population), n (%)			
Chronic pulmonary disease	1513 (100)	1314 (100)	2827 (100)
Diabetes without complications	485 (32.1)	428 (32.6)	913 (32.3)
Diabetes with complications	252 (16.7)	233 (17.7)	485 (17.2)
Peripheral vascular disease	259 (17.1)	221 (16.8)	480 (17.0)
Congestive heart failure	236 (15.6)	210 (16.0)	446 (15.8)
Renal disease	228 (15.1)	203 (15.4)	431 (15.2)
Blood eosinophil count, cells/µL, geometric mean (log SD)	379.4 (0.8)	388.2 (0.7)	383.5 (0.7)
Blood eosinophil count, n (%)			
0≤150 cell/ µL	0 (0)	0 (0)	0(0)
150–299 cells/µL	528 (34.9)	463 (35.2)	991 (35.1)
≥300 cells/µL	985 (65.1)	851 (64.8)	1836 (64.9)
Serum IgE, n (%)	219 (14.5)	315 (24.0)	534 (18.9)
Number of exacerbations, mean (SD)	2.74 (1.34)	2.93 (1.52)	2.83 (1.43)
Hospitalization-defined	0.08 (0.32)	0.10 (0.38)	0.09 (0.35)
ED-defined	0.35 (0.88)	0.37 (1.00)	0.36 (0.94)
Steroid-defined	2.31 (1.32)	2.45 (1.37)	2.38 (1.35)
≥1 biologic prescription, n (%)	79 (5.2)	258 (19.6)	337 (11.9)
Previous asthma treatments (≥25% of population), n (%)^‡^			
SABA/SAMA	1375 (90.9)	1190 (90.6)	2565 (90.7)
ICS only	573 (37.9)	501 (38.1)	1074 (38.0)
ICS/LABA	1454 (96.1)	1033 (78.6)	2487 (88.0)
Low dose	243 (16.7)	193 (18.7)	436 (17.5)
Medium dose	1074 (73.9)	331 (32.0)	1405 (56.5)
High dose	137 (9.4)	509 (49.3)	646 (26.0)
LAMA only	374 (24.7)	490 (37.3)	864 (30.6)
ICS/LABA/LAMA	388 (25.6)	430 (32.7)	818 (28.9)
LTRA	896 (59.2)	899 (68.4)	1795 (63.5)
OCS maintenance	564 (37.3)	668 (50.8)	1232 (43.6)
Payer, n (%)			
Commercial	511 (33.8)	438 (33.3)	949 (33.6)
Medicare	1000 (66.1)	876 (66.7)	1876 (66.4)
Unknown	2 (0.1)	0	2 (0.1)
Insurance plan type, n (%)			
Point of service	351 (23.2)	314 (23.9)	665 (23.5)
HMO	300 (19.8)	226 (17.2)	526 (18.6)
Other/unknown	862 (57.0)	774 (58.9)	1636 (57.9)

**Notes**: *Type 2 severe asthma was defined as being at GINA step 4 or 5 and having experienced ≥2 exacerbations with a blood eosinophil count of ≥150 cells/μL during the 12 months pre-index; ^†^Reported out of patients aged ≥35 years; ^‡^Patients could select more than one previous therapy option.

**Abbreviations**: CCI, Charlson comorbidity index; COPD, chronic obstructive pulmonary disease; CRS, chronic rhinosinusitis; ED, emergency department; GERD, gastro-esophageal reflux; GINA, Global Initiative for Asthma; HMO, health maintenance organization; ICS, inhaled corticosteroid; IgE, immunoglobulin E; LABA, long-acting β_2_-agonist; LAMA, long-acting muscarinic antagonist; LTRA, leukotriene receptor antagonist; OCS, oral corticosteroid; SABA, short-acting β_2_-agonist; SAMA, short-acting muscarinic antagonist; SD, standard deviation; USA, United States of America.

### Follow-Up Patient Characteristics

**Figure 2:**
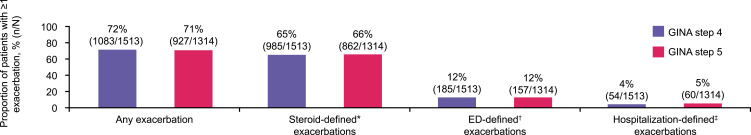
Exacerbations during the 12-month follow-up period in patients at GINA treatment steps 4 and 5. *Presence of any outpatient claims for OCS with an average daily dose of prednisone ≥20 mg (or equivalent) and days of supply of 3–28 days (inclusive) and a medical claim with a diagnosis of asthma in any position within ≥2 weeks of the OCS date or an injectable steroid claim with a diagnosis of asthma in any position within ≥2 weeks of the steroid date; ^†^Require a POS-23 or a CPT code between 99281 and 99288; ^‡^Presence of a confinement code on medical claim.

**Abbreviations**: CPT, Current Procedural Terminology; GINA, Global Initiative for Asthma; POS, place of service; OCS, oral corticosteroid; SD, standard deviation.

The proportions of patients with exacerbations during the 12-month follow-up period were similar between patients at both GINA steps (Figure 2 and eTable 2) and were primarily driven by systemic/oral steroid-defined exacerbations. Overall, similar proportions of patients at GINA treatment steps 4 and 5 (71.6% and 70.5%, respectively) experienced one or more exacerbations during the follow-up period. The mean (SD) total number of exacerbations was 1.6 (1.8) at GINA treatment step 4 versus 1.7 (1.9) at GINA treatment step 5. Mean (SD) steroid-defined exacerbations were 1.3 (1.5) versus 1.4 (1.6), ED-defined exacerbations were 0.2 (0.9) versus 0.2 (0.8), and hospitalization-defined exacerbations were 0.04 (0.3) and 0.06 (0.3) in the respective treatment groups. During the follow-up period, exacerbations (any type) occurred in 64.7% of GINA treatment step 5 patients who were receiving biologic therapies at baseline and in 72.0% of GINA treatment step 5 patients who were not receiving biologic therapies at baseline. The opposite was observed for patients at GINA treatment step 4; exacerbations occurred in a higher proportion of GINA treatment step 4 patients who were receiving biologic therapies at baseline compared with GINA treatment step 4 patients who were not receiving biologic therapies at baseline (78.5% vs 71.2%, respectively).

**Figure 3:**
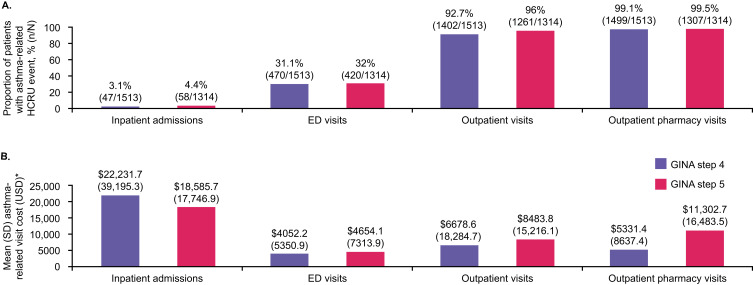
Asthma-related HCRU (**A**) and asthma-related costs (**B**) during the 12-month follow-up period in patients at GINA treatment steps 4 and 5. *Healthcare costs were summarized over the 12-month follow-up period and were adjusted to 2022 US dollars.

**Abbreviations**: ED, emergency department; GINA, Global Initiative for Asthma; HCRU, healthcare resource utilization; SD, standard deviation; USD, United States Dollar.

For all-cause HCRU in the overall population, 25.6% of patients underwent an inpatient admission, 54.4% underwent an ED visit, and nearly all patients (>99%) underwent outpatient and outpatient pharmacy visits (results not shown). Mean (SD) number of visits was 2.0 (1.8), 3.6 (4.9), 37.5 (30.2), and 39.7 (23.4) for inpatient admissions, ED visits, outpatient visits, and outpatient pharmacy visits, respectively. Slightly lower proportions of patients at GINA treatment step 4 experienced inpatient, ED or outpatient visits compared with patients at GINA treatment step 5; the proportion of patients undergoing outpatient pharmacy visits was slightly higher at GINA treatment step 4 versus 5 (Figure 3). For asthma-related HCRU in the overall population, 3.7% of patients underwent an inpatient admission, 31.5% underwent an ED visit, 94.2% underwent an outpatient visit, and >99% made an outpatient pharmacy visit; mean (SD) number of visits was 1.2 (0.6), 2.3 (2.5), 10.0 (13.2), and 16.4 (10.7), respectively. Similarly, a slightly lower proportion of patients at GINA treatment step 4 experienced asthma-related inpatient admissions or outpatient visits versus patients at GINA treatment step 5; the proportion of patients experiencing asthma-related ED or outpatient pharmacy visits was comparable. A lower proportion of patients at GINA treatment step 4 were readmitted compared with those at GINA treatment step 5 (34.0% vs 41.4%). This difference was most evident for readmissions within 90 days of discharge (19.1% vs 27.6%), as readmission rates within 30 days were similar for both GINA subgroups (circa 12% for both). Mean (SD) length of hospital stay in the overall population for patients who had an asthma-related inpatient admission (n=105) was 4.1 (2.9) days; the mean (SD) length of inpatient stay was 3.9 (3.0) and 4.3 (2.8) days for patients at GINA step 4 (n=47) and GINA step 5 (n=58), respectively.

All-cause and asthma-related total expenditure costs (mean [SD] and median [IQR]) are presented in Table 2, and key details relating to mean asthma-related costs are described below. Total asthma-related healthcare expenditure was lower at GINA treatment step 4 versus GINA treatment step 5, as was total asthma-related medical expenditure (Table 2). Asthma-related inpatient admission costs were the largest contributor to expenditure; mean costs were higher for GINA treatment step 4 versus 5 patients (median costs were comparable). All-cause and asthma-related inpatient admissions, ED visits, outpatient visits, and outpatient pharmacy visit data split by receipt of biologic therapy are shown in eTable 3. At GINA treatment step 5, a slightly higher proportion of patients receiving biologic therapies experienced asthma-related inpatient admissions or outpatient visits, while a lower proportion of patients experienced asthma-related ED visits compared with patients who did not receive biologic therapies. At GINA treatment step 4, patients receiving biologic therapies were more likely to experience asthma-related ED visits and outpatient visits but less likely to experience asthma-related inpatient admissions than their peers who did not receive biologic therapies. Notably, patients at GINA treatment step 5 who received biologics in the prior year had lower mean asthma-related inpatient costs compared with those who did not; median data were comparable. Only one patient at GINA treatment step 4 who received biologics experienced an asthma-related inpatient visit. Asthma-related outpatient visits and outpatient pharmacy costs were higher for patients who received biologic therapies in the prior year compared with those who did not. For asthma-related ED visits, both mean and median costs were higher for patients who received biologic therapies in the prior year compared with those who did not at GINA treatment step 4. Conversely, at GINA treatment step 5, results varied between mean and median costs; mean costs were higher for patients who received biologic therapies in the prior year compared with those who did not, while median costs were lower.

**Table 2:** All-Cause and Asthma-Related Healthcare Costs (US Dollars [2022]) During the 12-Month Follow-Up Period, Overall and by GINA Treatment Step

		Patients with Type 2 Severe Asthma* at GINA Step 4 (n=1513)	Patients with Type 2 Severe Asthma* at GINA Step 5 (n=1314)	Overall Population of Patients with Type 2 Severe Asthma* (n=2827)
Any all-cause inpatient admission costs	N (%)	372 (24.6)	352 (26.8)	724 (25.6)
	Mean (SD)	$50,027.1 (60,927.9)	$56,127.1 (72,568.8)	$52,992.9 (66,864.2)
	Median (Q1, Q3)	$29,007.7 (15,020.7, 58,291.9)	$31,171.1 (15,509.0, 60,408.4)	$30,353.1 (15,506.8, 58,670.0)
Any all-cause ED visit costs	N (%)	807 (53.3)	731 (55.6)	1538 (54.4)
	Mean (SD)	$6199.8 (9738.1)	$6583.6 (9952.6)	$6382.2 (9839.3)
	Median (Q1, Q3)	$3430.5 (1785.1, 7055.0)	$3750.8 (1867.8, 7351.4)	$3580.6 (1842.5, 7179.6)
Any all-cause outpatient visit costs	N (%)	1509 (99.7)	1314 (100)	2823 (99.9)
	Mean (SD)	$20,112.7 (30,936.3)	$23,080.5 (29,434.9)	$21,494.1 (30,277.6)
	Median (Q1, Q3)	$10,881.2 (4382.9, 24,508.4)	$12,965.3 (5164.2, 29,441.0)	$11,744.1 (4759.7, 27,016.3)
Any all-cause outpatient pharmacy costs	N (%)	1513 (100)	1313 (99.9)	2826 (>99.9)
	Mean (SD)	$9722.3 (16,474.8)	$15,785.4 (21,343.3)	$12,539.3 (19,130.6)
	Median (Q1, Q3)	$5069.5 (2585.2, 10,351.7)	$7688.5 (3949.3, 17,306.3)	$6006.9 (3094.1, 12,841.5)
Total all-cause medical expenditures	N (%)	1509 (99.7)	1314 (100)	2823 (99.9)
	Mean (SD)	$35,761.0 (57,391.1)	$41,778.7 (65,288.37)	$38,562.0 (61,256.5)
	Median (Q1, Q3)	$15,630.5 (5602.0, 42,398.3)	$19,340.1 (6691.1, 49,885.5)	$17,007.9 (5877.8, 45,745.7)
Total all-cause healthcare expenditures	N (%)	1513 (100)	1314 (100)	2827 (100)
	Mean (SD)	$45,388.8 (61,619.74)	$57,552.1 (70,913.8)	$51,042.3 (66,368.4)
	Median (Q1, Q3)	$24,024.37 (10,858.0, 54,978.3)	$34,571.5 (14,678.2, 72,484.5)	$28,513.0 (12,195.4, 63,127.4)
Any asthma-related inpatient admission Costs	N (%)	47 (3.1)	58 (4.4)	105 (3.7)
	Mean (SD)	$22,231.7 (39,195.3)	$18,585.7 (17,746.9)	$20,217.7 (29,248.0)
	Median (Q1, Q3)	$14,822.17 (11,292.5, 15,509.0)	$14,926.5 (14,054.8, 15,509.0)	$14,822.2 (11,374.4, 15,509.0)
Any asthma-related ED visit costs	N (%)	470 (31.1)	420 (32.0)	890 (31.5)
	Mean (SD)	$4052.2 (5350.9)	$4654.1 (7313.9)	$4336.2 (6356.7)
	Median (Q1, Q3)	$2376.3 (1556.3, 4703.9)	$2702.8 (1681.9, 5028.0)	$2541.8 (1556.3, 4867.0)
Any asthma-related outpatient visit costs	N (%)	1402 (92.7)	1261 (96.0)	2663 (94.2)
	Mean (SD)	$6678.6 (18,284.7)	$8483.8 (15,216.1)	$7533.4 (16,922.1)
	Median (Q1, Q3)	$1481.5 (577.3, 5147.9)	$2055.4 (751.4, 7852.6)	$1728.7 (650.9, 6188.2)
Any asthma-related outpatient pharmacy costs	N (%)	1499 (99.1)	1307 (99.5)	2806 (99.3)
	Mean (SD)	$5331.4 (8637.4)	$11,302.7 (16,483.5)	$8112.7 (13,237.2)
	Median (Q1, Q3)	$2933.3 (1333.4, 5281.7)	$4612.1 (2206.6, 11,121.7)	$3705.3 (1651.2, 7106.4)
Total asthma-related medical expenditures†	N (%)	1406 (92.9)	1263 (96.1)	2669 (94.4)
	Mean (SD)	$8757.4 (21,665.8)	$10,871.6 (18,484.4)	$9757.8 (20,246.6)
	Median (Q1, Q3)	$2042.8 (635.1, 8100.0)	$2906.3 (867.2, 11,700.9)	$2396.8 (701.5, 9385.8)
Total asthma-related healthcare expenditures	N (%)	1507 (99.6)	1313 (99.9)	2820 (99.8)
	Mean (SD)	$13,473.5 (24,836.0)	$21,708.6 (29,066.3)	$17,307.8 (27,195.8)
	Median (Q1, Q3)	$5897.9 (2930.9, 13,036.0)	$9383.0 (4724.7, 28,398.5)	$7306.2 (3613.1, 18,403.5)

**Notes**: *Type 2 severe asthma was defined as being at GINA step 4 or 5 and having experienced ≥2 exacerbations with a blood eosinophil count of ≥150 cells/μL during the 12 months pre-index. ^†^Medical expenditure was defined as the sum of inpatient, ED and outpatient expenditures, while healthcare expenditure was defined as the sum of inpatient, ED, outpatient and pharmacy expenditures.

**Abbreviations**: ED, emergency department; GINA, Global Initiative for Asthma; Q1, quartile 1; Q3, quartile 3; SD, standard deviation.

## Discussion

This study provides valuable insights into the real-world disease/economic burden and unmet treatment needs of patients with type 2 severe asthma, with a focus on describing variations across patients who received GINA treatment step 4 or 5 within the last 90 days. Patients at GINA treatment step 4 in this study were primarily managed with medium-dose ICS/LABA but were still experiencing asthma exacerbations. Relatively few patients at either GINA treatment step received biologic therapy during the baseline period; the underutilization of biologics is more pronounced among patients at GINA treatment step 4 compared with those at step 5. This may be driven by physician understanding of current GINA guidelines, as biologic use is suggested as an option at step 5 only,^1^ despite the fact that the patients at GINA treatment steps 4 and 5 in this study appear to share a similar burden of disease. Different perceptions and definitions of severity, with GINA step treatment being one potential definition (eg, vs exacerbation history), could also contribute to biologic underutilization.

Annual exacerbation rates were similar regardless of GINA step treatment received; systemic/oral steroid-defined exacerbations were reported most frequently, indicating that patients with type 2 severe asthma are at risk of increased cumulative steroid exposure-related side effects. HCRU was high across both GINA subgroups, with outpatient pharmacy visits representing the most frequent type of service used. Although inpatient admissions were less common, they contributed to the highest costs, highlighting a substantial healthcare burden in both groups. Exacerbations occurred more frequently in patients at GINA step 5 who were not prescribed biologics during baseline versus those who were prescribed biologics during baseline. The reverse was observed for GINA treatment step 4 patients (ie, those who were prescribed biologics during baseline were more likely to experience exacerbations than those who did not receive biologics), but the low number of patients included in this group (n=79) should be taken into consideration. Also, it should be noted that patients classed as being at GINA treatment step 4 and in receipt of biologic therapy were not receiving biologic therapy at the start of the study (ie, they had received biologics prior to study start, during the baseline period). These patients might have been at higher risk than their peers of experiencing uncontrolled asthma; this is supported by the baseline exacerbation history data (ie, a higher proportion of patients at GINA treatment step 4 who received biologic therapy experienced hospitalization/ED-defined exacerbations compared with patients at GINA treatment step 4 who did not receive biologic therapy).

The cost–benefit profile of severe asthma treatment should be considered. Patients who received biologic therapies at GINA treatment step 5 (n=258) experienced slightly more asthma-related inpatient admissions than those who did not receive such therapies, but were less likely to undergo ED visits. In terms of the associated visit costs, patients at GINA treatment step 5 who received biologic therapies recorded lower mean asthma-related inpatient admission costs than patients who did not, while the reverse was shown for mean asthma-related ED visit, outpatient visits, and outpatient pharmacy costs at both GINA treatment steps. It should be noted that mean and median results differed in some cases, making interpretation of these results challenging.

Although short-term management with OCS is often considered to be a low-cost option, OCS use has been linked to an increased risk of long-term complications which may contribute to higher overall healthcare costs in the long term and also fails to address the underlying chronic inflammation characteristic severe asthma.^22^,^23^ While initially expensive, biologic use has the potential to improve long-term patient outcomes and reduce complications, thus reducing exacerbations and consequent HCRU.^11^ It should be noted that data on duration of biologic treatment was not collected in this study (patients were listed as having received biologic treatment within the past 12 months), so patients in receipt of biologic treatment may have only received such treatment for a brief period of time and then discontinued therapy. It should be noted that previous studies have indicated significant improvements in exacerbation rate and other outcomes at 1 year of treatment.^8^,^12^,^13^,^24^

These results are in line with those reported in other studies, including a systematic literature review of patients with severe asthma^25^ and a database study including US-based patients with severe asthma who received biologic therapy.^26^ In those studies, uncontrolled asthma (indicated by ED, outpatient and physician visit data) was associated with increased costs.^25^,^26^

This study utilized data from an administrative database of patients with asthma across the US, allowing detailed characterization of exacerbation frequency, HCRU, and costs among patients with severe asthma. This provides valuable oversight of the disease burden, covering both clinical and economic aspects, of patients with frequently exacerbating asthma characterized by blood eosinophils (a subgroup often too small in proportion to be adequately studied). However, there were some limitations that should be noted. Firstly, the patient population were mostly aged over 60 years, predominantly female and tended to have comorbid conditions, with the majority receiving Medicare rather than commercial insurance. These factors may have impacted the generalizability of these results. In particular, the high degree of comorbidity (including COPD) suggests a mixed airways disease population. While inclusion of these patients reflects the real-world asthma population and strengthens generalizability of the study, it should be acknowledged that it may impact on exacerbations and costs compared with an asthma-only population. Secondly, it is possible that confounding by indication may be present, particularly when comparing biologic users and non-users. Since patients who received biologics may be a clinically distinct subgroup, these results should be interpreted cautiously. Next, as previously described, patients were categorized into GINA treatment steps based on their asthma treatments in the 90 days prior to the index date. A total of 75 patients who were prescribed a biologic during the 1-year baseline period discontinued the biologic within this 90-day period and were thus classified as GINA step 4; this may have potentially influenced the outcomes for both treatment step groups and also raises the potential for GINA misclassification. On a similar note, changes in background therapy may have had an impact on exacerbations and HCRU, as patients may have previously received high-dose ICS/LABA and stepped down to medium-dose ICS/LABA at the time this study was conducted. Data from a real-world historic cohort study indicate that stepping up to high-dose ICS/LABA does not prevent future asthma exacerbations,^27^ supporting the potential utility of biologic therapy for GINA step 4 patients on medium-dose ICS/LABA who are experiencing exacerbations. Also, asthma-related HCRU and costs may have been overestimated due to the study approach of capturing asthma claims in any setting, with the consequence that not all costs may have been directly attributable to asthma. Due to potential coding or billing inaccuracies in the diagnosis codes, there remains potential for misclassification of asthma-related HCRU measures. In addition, because non-usage was assumed and assigned zero for cost summaries in cases where a patient lacked a record of service (inpatient, outpatient, ED, outpatient pharmacy), it should be noted that this assumption may not always apply and therefore may have introduced a potential underestimation of costs. Furthermore, prior biologic use may have been for other indications than asthma, or not captured due to clinical trial participation which may have contributed to data bias due to the potential misclassification of indications related to imperfect coding or misdiagnosis. Finally, it should be noted that all comparisons were observational; therefore, no causal inferences should be drawn.

## Conclusions

Patients with type 2 severe asthma receiving treatment at GINA steps 4 and 5 experience similar levels of disease burden and comparable annual exacerbation rates. These rates were largely driven by OCS bursts, contributing to increased cumulative OCS exposure and associated side effects. Currently, patients are often required to step through escalating GINA treatment regimens, including a transition to high-dose ICS/LABA, before being considered for biologic therapy. This step-up approach may delay effective intervention, thereby increasing the risk of exacerbations and OCS-related complications. In the current study, similar asthma-related HCRU was observed between GINA treatment step 4 and 5 patients. Biologic-naïve patients at GINA treatment step 5 had more recurrent exacerbations than those previously treated with biologics, yet only 20% of eligible patients at GINA treatment step 5 were receiving biologics, highlighting an unmet treatment need. These findings highlight a potential unmet need for earlier phenotypic assessment in patients at GINA treatment step 4, given the similarly high disease burden to GINA treatment step 5 observed in this group. Patients in this population experiencing multiple exacerbations (or who are at future risk of exacerbations) should ideally undergo early phenotyping for biologic eligibility and subsequent targeted asthma treatment, with the aim of improving patient outcomes and reducing the clinical and economic burden of severe asthma.

## Data Availability

Please refer to GSK weblink to access GSK’s data sharing policies and as applicable seek anonymized subject-level data via the link: https://www.gsk-studyregister.com/en/.

## Abbreviations

CBC, complete blood count; CDM, Clinformatics^®^ Data Mart; ED, emergency department; GERD, gastroesophageal reflux disease; GINA, Global Initiative for Asthma; HCRU, healthcare resource utilization; HIPAA, Health Insurance Portability and Accountability Act; ICS, inhaled corticosteroid; ICD-10-CM, International Classification of Diseases, Tenth Revision, Clinical Modification; IL, interleukin; LABA, long-acting β_2_-agonist; LAMA, long-acting muscarinic antagonist; LTRA, leukotriene receptor antagonist; OCS, oral corticosteroids; SABA, short-acting β_2_-agonist; SAMA, short-acting muscarinic antagonist; SAS, Statistical Analysis Software; SD, standard deviation; USA, United States.
